# The effect of ovarian stimulation protocol on follicular asynchrony during in vitro fertilization – A retrospective cohort study

**DOI:** 10.1002/ijgo.71186

**Published:** 2026-06-24

**Authors:** Alyssa Hochberg, Maria Hincapie, Uri Amikam, Nir Kugelman, William Buckett, Shauna Reinblatt

**Affiliations:** ^1^ Department of Obstetrics and Gynecology McGill University Quebec Canada; ^2^ The Faculty of Medicine Tel Aviv University Tel Aviv Israel; ^3^ Rappaport Faculty of Medicine Technion‐Israel Institute of Technology Haifa Israel

**Keywords:** controlled ovarian stimulation, follicular asynchrony, follicular maturation rate, IVF protocol, ovum pick‐up

## Abstract

**Objective:**

To examine the effect of controlled ovarian stimulation (COS) protocol on follicular synchrony in a subsequent in vitro fertilization (IVF) cycle in patients whose previous cycle was canceled due to asynchrony.

**Methods:**

A single‐center retrospective‐cohort study (January 2018–March 2024), including women undergoing IVF who had: (1) a COS cycle canceled without oocyte‐pick‐up (OPU) due to follicular asynchrony; and (2) a subsequent COS cycle with OPU performed. Patients were grouped as: Group 1–same protocol repeated; Group 2‐protocol switched. Follicular asynchrony was defined as ≤50% of stimulated follicles ≥14 mm at cancellation. Women with ≤3 total stimulated follicles at cancellation were excluded. Follicular maturation rate in each cycle was the proportion of follicles ≥14 mm among all stimulated follicles at cancellation/trigger. Primary outcome was the difference in follicular maturation rate between the OPU and canceled cycles, representing synchrony improvement. Treatment outcomes were compared; analysis of variance (ANOVA) controlled for confounders.

**Results:**

Overall, 95 patients met the inclusion criteria: 43 (45.26%) in Group 1, and 52 (54.74%) in Group 2. Group 1 versus Group 2 had higher parity and antral follicle count (AFC), and lower endometriosis prevalence and daily recombinant‐luteinizing‐hormone (rLH) dose in the OPU cycle (*p* < 0.05). Follicular maturation rate difference was significantly lower in Group 1 versus Group 2 (0.17 [0.05–0.28] vs. 0.3 [0.15–0.39], *p* = 0.03). Utilizing ANOVA adjusting for group, parity, endometriosis, daily rLH dose in the OPU cycle, and AFC, only group designation remained significantly associated with improved follicular maturation, with increased improvement in Group 2 (least squares mean 0.27 vs. 0.12, *p* = 0.01).

**Conclusion:**

Switching COS protocols after a cycle canceled for asynchrony was associated with improved follicular synchrony, suggesting consideration for protocol modification in a subsequent cycle.

## 1 INTRODUCTION

One of the primary objectives of controlled ovarian stimulation (COS) for in vitro fertilization (IVF) is achieving optimal follicular growth in order to collect the maximum number of mature oocytes. During the early follicular phase, early antral follicles may present with markedly different sizes, and this finding may be amplified during COS. When fewer follicles contain mature oocytes, there are fewer viable oocytes and therefore fewer embryos available for selection for transfer.^1^ Whereas the underlying mechanism of antral follicle size heterogeneity remains unclear, one possible explanation involves the exposure of early antral follicles to gradient follicle‐stimulating hormone (FSH) levels during the previous luteal phase.^2^ To further complicate matters, there is no accepted definition of follicular asynchrony in the IVF literature.^1^, ^3^, ^4^ In women with polycystic ovary syndrome (PCOS), this phenomenon is particularly relevant due to the high antral follicle count and variable follicular sensitivity to FSH, which can result in suboptimal ovarian response and lower live birth rates.^5^, ^6^ As a result, the best approach to improve follicular synchrony in a subsequent COS cycle remains unknown. In clinical practice, the question arises whether the COS protocol should be changed in a subsequent cycle. Regarding the definition of follicular asynchrony, since follicles with a mean diameter of ≥ 14 mm are estimated in the medical literature to contain a mature oocyte, and this threshold is used to guide the timing of ovulation triggering and oocyte retrieval,^7^, ^8^, ^9^, ^10^, ^11^, ^12^, ^13^, ^14^ we chose to define follicular asynchrony in the canceled cycle when ≤ 50% of stimulated follicles reached a mean diameter of ≥ 14 mm at the time of cancellation decision, which is the definition used at our center to guide decision making. This essentially represents a surrogate marker for oocyte maturity, exemplifying the ‘presumed’ rate of mature oocytes out of the total number retrieved, a marker unavailable for analysis when an oocyte pick‐up (OPU) is canceled.

Due to varying funding programs for IVF worldwide, some clinics, including our own, allow for only one funded IVF COS cycle. In such cases, a single IVF cycle cancellation with continued funding for a subsequent cycle is allowed, in the event of a predicted suboptimal response to COS in the first cycle. Along these lines, cycles are sometimes canceled due to follicular asynchrony (as decided by the treating physicians), with a subsequent cycle proceeding to an OPU regardless of the COS results, since this is the only chance for a funded cycle. In such cases, the question arises whether the COS protocol should be modified in the subsequent cycle. Hence, the aim of our current study was to examine the effect of COS protocol on follicular asynchrony in the subsequent stimulation cycle, in patients presenting with cycle cancellation for follicular asynchrony in the preceding cycle.

## 2 MATERIALS AND METHODS

### 2.1 Study design and participants

We conducted a single‐center retrospective cohort study including women who underwent a fresh IVF stimulation cycle (2018–2024). We included all patients who had: (1) their initial OPU canceled due to follicular asynchrony and (2) underwent a subsequent cycle with an OPU performed. The sample size was determined by all eligible patients during the study period. Patients were divided into groups according to the COS protocol utilized in the subsequent cycle: Group 1‐those receiving the same protocol; Group 2‐patients whose protocol was switched.

Following COS, follicles with a mean diameter of ≥ 14 mm were estimated to contain a mature oocyte; as such, we defined follicular asynchrony (a surrogate marker for oocyte maturity) in the canceled cycle when ≤50% of stimulated follicles reached a mean diameter of ≥ 14 mm at the time of trigger. Women with <4 mature total stimulated follicles prior to cycle cancellation decision were excluded.

### 2.2 Fresh IVF stimulation protocols

The fresh IVF stimulation regimens were determined by the patient's treating physician, taking into consideration her age, ovarian reserve parameters, and prior COS response (where applicable). Three standard COS protocols were employed: (1) the GnRH‐antagonist protocol, (2) the long GnRH‐agonist protocol and (3) the short‐GnRH agonist “flare” protocol. None of the participating patients were treated with oral contraceptive pills or any other hormonal contraception prior to COS. Patients underwent a baseline vaginal ultrasound examination on days 2–3 of their menstrual cycle by trained physicians/technicians. COS was initiated if the endometrial lining was < 6 mm and the ovarian follicles measured < 10 mm in mean diameter. At our center, it is standard to measure and record all follicles prior to and during COS, regardless of their size (including those < 10 mm). Patients received daily subcutaneous injections of either highly purified human menopausal gonadotropins (hMG), recombinant‐FSH, recombinant‐FSH combined with hMG, or recombinant‐FSH combined with recombinant‐LH. Daily FSH doses ranged from 150 to 450 IU. Ovarian response was monitored using serial transvaginal ultrasonography and serum estradiol measurements, with gonadotropin doses adjusted as required. When ≥ 3 follicles reached 17 + mm in diameter, patients received subcutaneous injections of either recombinant‐hCG (250 mcg) or GnRH agonist (1 mg buserelin), or both. Oocyte retrieval was performed by transvaginal ultrasound‐guided puncture of follicles 35–37 h after the trigger injection. The follicular fluid aspirated was analyzed in the IVF laboratory, and the total number of retrieved oocytes was recorded.

### 2.3 Data collection

Data encompassed patient demographics, infertility cause(s), testing results, and cycle characteristics.

### 2.4 Outcomes

The follicular maturation rate in each cycle (canceled and subsequent OPU) was defined as the rate of follicles ≥ 14 mm out of all stimulated follicles at the time of cycle cancellation/trigger decision, respectively. Primary outcome was follicular maturation rate difference, defined as the difference between the follicular maturation rate in the OPU cycle and the rate in the canceled cycle, representing follicular synchrony improvement. Treatment outcomes were compared between the two groups.

### 2.5 Statistical analysis

Categorical data are presented by the number of cases and percentages, and continuous data using the median and interquartile range (IQR). Categorical data were subjected to Pearson Chi‐square analysis and Fisher exact test, and continuous data underwent the student's *t*‐test and Wilcoxon test as appropriate. Analysis of variance (ANOVA) was utilized to control for the potential confounding effects of pre‐existing clinical characteristics, maternal demographics, and concurrently occurring conditions where *P* values were less than 0.05 on the chi‐squared tests. The assumptions underlying ANOVA were assessed prior to analysis. Normality of residuals was evaluated using the Shapiro–Wilk test and visual inspection of Q–Q plots, and homogeneity of variances was assessed using Levene's test. These assumptions were met for the variables analyzed. As this was a retrospective study, no a priori sample size calculation was performed. However, the sample size was determined by all eligible patients during the study period. Computations were performed using SAS software (version 9.4: Cary, North Carolina, USA).

### 2.6 Ethics

The study received approval from the ethics committee of the McGill University Health Center (MUHC) Reproductive Center (MUHC REB 2024‐10599). Informed consent was waived due to the study's retrospective design.

## 3 RESULTS

### 3.1 Cohort characteristics

Overall, 95 patients met the inclusion criteria: 43 (45.26%) in Group 1 (“same protocol” group), and 52 (54.74%) in Group 2 (“different protocol” group). Table 1 presents the baseline, treatment and cycle characteristics of both groups. As depicted, patients in Group 1, compared to Group 2, were characterized by higher median parity (0 [IQR: 0–1] vs. 0 [0–0], *p* = 0.02) and antral follicle count (AFC) (15 [10–27] vs. 12 [6–18], *p* = 0.032); a lower rate of endometriosis (2.33% vs. 17.31%, *p* = 0.02); and a lower median recombinant‐luteinizing hormone (rLH) daily dose in the OPU cycle (150 [150–225] vs. 225 [150–300] IU, *p* = 0.01). Other baseline and cycles characteristics were comparable between groups, including serum AMH values.

**TABLE 1:** Baseline, treatment, and cycle characteristics of the study cohort.

Variable	Group 1—same protocol (*n* = 43)	Group 2—different protocol (*n* = 52)	*p*
Maternal age at canceled cycle (years)	36.11 (32.4–39.3)	36.95 (34.75–39.25)	0.17
Maternal age at next OPU cycle (years)	37 (32.6–39.11)	37.15 (35.16–39.55)	0.21
Months elapsed between canceled cycle and next OPU cycle	2 (2–4)	2 (2–3)	0.59
Canceled stimulation cycle number	1 (1–2)	1 (1–1)	0.06
BMI	23.87 (22–26.95)	23.5 (21–26.84)	0.56
Gravidity	1 (0–2)	0 (0–1)	0.054
Parity	0 (0–1)	0 (0–0)	**0.02**
Previous cesarean delivery	4 (9.3)	5 (9.62)	1.0
Previous miscarriage	13 (30.23)	13 (25)	0.65
Smoking	0 (0)	2 (3.85)	0.5
Fibroids	4 (9.3)	12 (23.08)	0.1
Adenomyosis	1 (2.33)	1 (1.92)	1.0
Thyroid disorder	6 (13.95)	5 (9.62)	0.54
Hyperprolactinemia	1 (2.33)	0 (0)	0.45
Ovulatory disorder	4 (9.3)	7 (13.46)	0.75
Male factor	27 (62.79)	27 (51.92)	0.3
Tubal factor	5 (11.63)	9 (17.31)	0.57
Advanced maternal age (≥ 40 years)	6 (13.95)	11 (21.15)	0.43
Unexplained infertility	5 (11.63)	6 (11.54)	1.0
Diminished ovarian reserve	17 (39.53)	23 (44.23)	0.68
Endometriosis	1 (2.33)	9 (17.31)	**0.02**
Preimplantation genetic testing	3 (6.98)	2 (3.85)	0.66
Egg donor	0 (0)	1 (1.92)	1.0
Uterine factor	1 (2.33)	2 (3.85)	1.0
Infertility type			0.39
Primary	26 (60.47)	35 (70)^a^	
Secondary	17 (39.53)	15 (30)^a^	
Infertility duration (months)	30 (12–36)	26 (18–39)	0.97
Antral follicle count	15 (10–27)	12 (6–18)	**0.032**
Anti‐Mullerian hormone (ng/mL)	1.54 (0.82–3.16)	1.4 (0.56–2.71)	0.16
Fertility preservation	0 (0)	2 (3.85)	0.5
Canceled cycle characteristics			
Daily FSH dose in the canceled cycle (IU)	275 (200–330)	300 (225–450)	0.11
Days of stimulation in the canceled cycle	8 (7–9)	8 (7–9)	0.9
Daily LH dose in the canceled cycle (IU)	150 (75–150)	150 (75–225)	0.16
Peak estradiol level in the canceled cycle (pmol/L)	2624 (1706–4030)	2054 (1498–2777)	0.1
Peak endometrial thickness in the canceled cycle (mm)	9.45 (8.4–11)	9.7 (7.5–11.2)	0.8
Number of follicles ≥ 14 mm in the canceled cycle	2 (2–3)	2 (1–2)	**0.01**
Number of follicles < 14 mm in the canceled cycle	6 (4–7)	5 (4–7)	0.71
Total number of stimulated follicles in the canceled cycle	8 (5–11)	7 (5–9.5)	0.29
Follicular maturation rate in the canceled cycle	0.29 (0.23–0.4)	0.21 (0.16–0.37)	**0.03**
Largest follicle size in the canceled cycle (mm)	19 (18–20)	19 (18–21)	0.61
Canceled cycle protocol type			0.2
GnRH antagonist	36 (83.72)	45 (86.54)	
Micro‐dose GnRH agonist flare	3 (6.98)	0 (0)	
Long GnRH agonist	4 (9.3)	7 (13.46)	
OPU cycle characteristics			
OPU cycle protocol type			**<0.001**
GnRH antagonist	36 (83.72)	7 (13.46)	
Micro‐dose GnRH agonist flare	3 (6.98)	19 (36.54)	
Long GnRH agonist	4 (9.3)	26 (50)	
Daily FSH dose in the OPU cycle (IU)	405 (300–450)	450 (300–450)	0.1
Days of stimulation in the OPU cycle	9 (8–10)	11 (9–12)	**0.001**
Daily LH dose in the OPU cycle (IU)	150 (150–225)	225 (150–300)	**0.01**

*Note*: BMI, calculated as weight in kilograms divided by the square of height in meters. Continuous variables are presented as median (IQR) and categorical variables are presented as *n* (%). Bold values indicate statistical significance (*p* < 0.05).

Abbreviations: BMI, body mass index; FSH, follicle‐stimulating hormone; GnRH, gonadotropin‐releasing hormone; IQR, interquartile range; LH, luteinizing hormone; OPU, ovum pick‐up.

^a^ Two patients in this group did not have an infertility diagnosis, but rather one was an egg donor and one underwent a fertility preservation cycle.

### 3.2 IVF stimulation cycle outcomes

Table 2 depicts the IVF stimulation cycle outcomes in both groups. In the univariate analysis, follicular maturation rate difference was significantly lower in Group 1 as compared to Group 2 (0.17 [0.05–0.28] vs. 0.3 [0.15–0.39], *p* = 0.03). Additionally, in both groups, some subsequent OPU cycles retained follicular asynchrony regardless of treatment protocol group designation, as displayed by the follicular maturation rate in the OPU cycle (0.46 [0.38–0.6] in Group 1 vs. 0.5 [0.4–0.6] in Group 2, *p* = 0.35).

**TABLE 2:** In vitro fertilization stimulation cycle outcomes of the study cohort.

Variable	Group 1—same protocol (*n* = 43)	Group 2—different protocol (*n* = 52)	*p*
Peak estradiol level in the OPU cycle (pmol/L)	4321 (2955–6500)	4274 (2679–6697)	0.66
Peak endometrial thickness in the OPU cycle (mm)	9.9 (9.1–12.1)	9.85 (8–12)	0.34
Number of follicles ≥ 14 mm in the OPU cycle	5 (3–7)	5 (2.5–7)	0.17
Number of follicles < 14 mm in the OPU cycle	5 (3–11)	4 (2–8)	0.08
Total number of stimulated follicles in the OPU cycle	11 (7–17)	10 (5–15)	0.08
Largest follicle size in the OPU cycle (mm)	20 (19–21)	20 (18–21)	0.2
Follicular maturation rate in the OPU cycle	0.46 (0.38–0.6)	0.5 (0.4–0.6)	0.35
Follicular maturation rate difference (OPU cycle minus the canceled cycle)	0.17 (0.05–0.28)	0.3 (0.15–0.39)	**0.03**

*Note*: Continuous variables are presented as median (IQR) and categorical variables are presented as *n* (%). Bold values indicate statistical significance (*p* < 0.05).

Abbreviations: IQR, interquartile range; OPU, ovum pick‐up.

### 3.3 ANOVA

Utilizing ANOVA adjusting for group, parity, endometriosis, daily rLH dose in the OPU cycle, and AFC, the only factor found to be associated with improved follicular maturation was group designation, with increased improvement in the “different protocol” group (least squares mean 0.27 vs. 0.12, *p* = 0.01).

## 4 DISCUSSION

### 4.1 Main findings

We aimed to evaluate the effect of COS protocol on follicular synchrony in patients whose IVF cycles were canceled due to follicular asynchrony. Utilizing ANOVA adjusting for confounders, the only factor found to be associated with improved follicular maturation was group designation, with improvement seen in the group whose subsequent cycle utilized a different COS protocol. Additionally, in both groups, some patients retained follicular asynchrony in the subsequent OPU cycle.

### 4.2 Interpretation

Achieving coordinated multiple follicular growth during COS for IVF is one of the main objectives of treatment, aiming to optimize mature oocyte retrieval. However, follicular asynchrony, that is, dis‐coordinated follicular growth, is not well‐defined in the literature nor in clinical practice and presents a challenge for treating physicians. Due to the lack of a clear definition, its frequency and treatment adjustments aimed to minimize asynchrony are difficult to assess. Some studies define follicular asynchrony during COS for IVF as the presence of ≥ 1 leading follicles ≥ 4 mm larger than the remaining cohort, with this being associated with reduced oocyte yield, impaired embryo quality, and lower implantation rates.^5^ In the present study, we aimed to define a measure of follicular asynchrony, referring to the chance that a stimulated follicle would contain a mature oocyte. As such, follicles with a mean diameter of ≥ 14 mm were estimated to contain a mature oocyte, in accordance with the literature setting this threshold to guide the timing of ovulation triggering and oocyte retrieval.^7^, ^8^, ^9^, ^10^, ^11^, ^12^, ^13^, ^14^ Accordingly, follicular asynchrony in the canceled cycle was defined when ≤ 50% of stimulated follicles reached a mean diameter of ≥ 14 mm at the time of cancellation decision. Since follicular asynchrony following COS for IVF directly impacts the number of retrieved oocytes and subsequent pregnancy rates,^1^ it is important to understand how to optimize treatment outcomes in such cases.

After controlling for potential confounders, our findings demonstrate an improved follicular maturation rate when the subsequent COS protocol was switched (most frequently from a short to a longer protocol). Inherent biological fluctuations leading to intercycle variability in the ovarian response to COS for IVF in the same patient are well‐known,^15^, ^16^, ^17^, ^18^, ^19^, ^20^ and affected by many factors. These include patient‐specific factors, such as body mass index (BMI, calculated as weight in kilograms divided by the square of height in meters), genetics, ovarian reserve parameters (basal FSH, AFC, and anti‐Mullerian hormone [AMH]), and the patient's FSH‐receptor genotype,^19^ in addition to hormonal fluctuations and follicular readiness. Other important factors include stimulation related parameters, such as COS protocol, gonadotropin daily dose and type, as well as number of stimulation days, trigger type and OPU practice. When considering biological factors affecting ovarian response to COS, these include variations in the antral follicle cohort available for recruitment by gonadotropins, and variability of ovarian follicular sensitivity to COS. Since inherent biological fluctuations are difficult to assess, our findings provide a practical tool to try and improve follicular synchrony by selecting an appropriate COS protocol in the subsequent cycle. Looking forward, patient and treatment specific factors may be amenable for implementation in future artificial intelligence models, to better select the COS protocol resulting in improved follicular synchrony.

It has been shown that switching IVF stimulation protocols between cycles (regardless of follicular synchrony in the earlier cycle) can alter oocyte yield, though the effect depends on the specific protocols and patient characteristics. For example, mild stimulation yields fewer oocytes (median 6) than conventional protocols (median 9) in meta‐analyses and randomized trials,^21^, ^22^, ^23^ yet still achieves optimal implantation rates.^22^, ^23^ Switching protocols has generally not been shown to improve laboratory outcomes; a large retrospective cohort study found slightly better oocyte numbers and usable embryos when repeating the same protocol (except flare), while switching did not consistently increase oocyte yield.^24^ Certain targeted modifications—such as adding a 3‐day antagonist pretreatment—have increased oocyte recovery (from 7.8 to 10 oocytes) in subsequent cycles.^25^ Thus, oocyte yield can change when switching COS protocols, but the direction and magnitude of change depend on the protocols compared and patient factors. Protocol selection should be tailored to optimize both oocyte yield and clinical outcomes.^21^, ^22^, ^23^, ^24^, ^25^ Unlike prior studies, our study focuses specifically on cycles canceled for follicular asynchrony and compares the canceled cycle with the subsequent OPU cycle using follicular maturation rate as a proxy for true mature oocyte yield. This comparison has not been reported previously and may help guide protocol decisions after a canceled cycle, potentially reducing costs and avoiding an unnecessary invasive procedure with sub‐optimal results. Regarding proposed mechanisms for follicular synchrony improvement, long GnRH agonist protocols are theorized to increase follicular synchronicity through prolonged pituitary downregulation, which may reduce discrepancy in follicle growth and result in a more homogenous cohort, potentially improving oocyte yield.^26^ In contrast, GnRH antagonist protocols are thought to produce more asynchronous follicle growth due to uneven baseline follicle size in the absence of prior pituitary downregulation.^26^ It should be noted, however, that Group 2 was heterogeneous, encompassing a range of specific protocol modifications rather than a single uniform change. This limits our ability to determine which particular protocol adjustments are most beneficial for improving follicular synchrony, and future prospective studies should aim to evaluate the comparative effectiveness of specific protocol combinations in this setting.

A recent study suggested that individualized dosing of recombinant‐FSH, particularly with follitropin delta, can significantly reduce the incidence of both early and late follicular asynchrony in women with PCOS undergoing IVF. In a matched case–control study, individualized dosing (based on AMH and bodyweight) resulted in lower rates of asynchrony (defined when 1–2 leading follicles were at least 4 mm larger in diameter than the rest of the cohort on days 5 and 9 of stimulation) compared to standard dosing.^5^ Conversely, in our study, daily FSH dose in the OPU cycle was comparable between groups, thus not affecting our results.

We demonstrated that in both groups, some cycles retained follicular asynchrony in the subsequent OPU cycle. This may be due to patient‐specific factors, including age, BMI, ovarian reserve markers (such as AFC, AMH, and baseline FSH levels), and the underlying infertility cause (e.g., PCOS). Age is a critical determinant, with older women more likely to experience asynchronous follicular growth due to diminished ovarian reserve and altered follicular dynamics.^27^, ^28^, ^29^ Higher BMI and elevated baseline FSH are associated with poorer and more asynchronous ovarian responses, while higher AMH predicts a greater risk of hyper‐response and asynchrony.^19^ Women with PCOS are also at increased risk for follicular asynchrony due to their high AFC and heterogeneous follicular sensitivity to FSH.^5^ Lastly, genetic factors, such as FSH receptor polymorphisms, and previous ovarian response history further modulate individual susceptibility to asynchrony.^18^, ^30^

### 4.3 Clinical implications

Follicular asynchrony is clinically significant because it is associated with fewer mature oocytes retrieved, reduced embryo quality, and decreased implantation rates.^5^ Suboptimal ovarian response, often characterized by delayed follicular development and lower estradiol/progesterone levels in the late follicular phase, correlates with lower live birth rates in both fresh and cumulative cycles.^31^, ^32^, ^33^, ^34^

Regarding the clinical relevance of the observed additional 0.13 follicular maturation rate difference in Group 2 (0.30) as compared to Group 1 (0.17), reflecting greater improvement in follicular synchrony in Group 2, this corresponds to approximately 13% more recruited follicles reaching estimated maturity. Depending on the number of oocytes retrieved (acknowledging follicular maturation rate as a proxy measure), this may translate into a proportional increase in the number of mature oocytes per OS cycle. The clinical importance of this finding lies in the observation that the cumulative live birth rate increases progressively with each additional mature oocyte retrieved, with the strongest gains observed up to approximately 15–25 mature oocytes, after which the benefit plateaus.^35^, ^36^, ^37^, ^38^, ^39^, ^40^, ^41^

### 4.4 Strengths and limitations

Our study had several strengths. First, its novelty lies in defining follicular maturation rate, thereby enabling evaluation of the effect of COS protocol modification in a subsequent cycle on follicular maturation, even when a previous COS cycle has been canceled (rather than assessing the difference in retrieved or mature oocyte yield). Second, our study's single‐center design ensured similar follicular follow‐up and measurements by the same physicians'/technicians' strategies, enabling a more accurate comparison between groups.

Our study had some limitations. First, due to its retrospective nature, some data may be missing, potentially affecting our results, and the non‐random allocation of protocol change limits causal inference. Additionally, residual confounding from unmeasured variables cannot be excluded, and the OS protocol selected by the treating physician may represent an additional source of confounding. Second, due to our single‐center design, our findings may not be generalizable to other centers. Third, due to the limited number of cases and to enhance statistical power, we analyzed OS protocols collectively. Although protocol types in the canceled OPU cycle did not differ significantly between groups, outcomes may differ across specific protocol combinations. Fourth, due to our small sample size, we may have been underpowered to detect additional differences between groups, if such exist. Nevertheless, due to the varying practice worldwide regarding COS cycle cancellation due to follicular asynchrony, our effort to define follicular asynchrony and evaluate the effect of subsequent COS protocol modification on follicular maturity (as opposed to retrieved or mature oocyte yield), may aid physicians facing such challenges when choosing the subsequent COS protocol, and serve as a benchmark for conducting future large prospective studies. These studies should also aim to elucidate more clearly the utility of protocol adjustment in subsequent cycles when utilizing specific protocol combinations. Lastly, although we assessed follicular maturation rate, we acknowledge that our definition is not universal, and that it is not a validated clinical endpoint. In the absence of OPU in the first cycles, this measure serves only as a proxy outcome and may not fully reflect mature oocyte yield. Because the first OPU was canceled, direct comparison of oocyte or mature oocyte yield between the canceled and subsequent OPU cycles was not possible, making our endpoint a surrogate marker for oocyte maturation rate, with limited predictive ability on embryo quality, pregnancy rates or live birth rates.

## 5 CONCLUSION

We found that switching the COS protocol in the subsequent IVF cycle was associated with improved follicular maturation when the previous COS cycle was canceled due to follicular asynchrony, although some degree of asynchrony remained. As our primary outcome is a surrogate marker for oocyte maturation rate—not a validated clinical endpoint—these findings should be interpreted with caution, and clinical implications regarding embryo quality, pregnancy rates, or live birth rates cannot be directly inferred. With this important limitation in mind, protocol modification could cautiously be considered in subsequent cycles following cancellation for follicular asynchrony.

## AUTHOR CONTRIBUTIONS

AH: Conceptualization, data curation, formal analysis, investigation, methodology, project administration, resources, software, supervision, validation, visualization, writing – original draft, writing – review and editing. MH: Data curation, formal analysis, investigation, methodology, project administration, resources, writing – review and editing. UA: Data curation, formal analysis, investigation, methodology, project administration, resources, software, supervision, validation, visualization, writing – review and editing. WB: Conceptualization, data curation, formal analysis, investigation, methodology, project administration, resources, software, supervision, validation, visualization, writing – original draft, writing – review and editing. SR: Conceptualization, data curation, formal analysis, investigation, methodology, project administration, resources, software, supervision, validation, visualization, writing – original draft, writing – review and editing.

## FUNDING INFORMATION

This research received no specific grant from public, commercial, or not‐for‐profit funding agencies.

## CONFLICT OF INTEREST STATEMENT

The authors have no conflict of interests to declare.

## Data Availability

All data supporting the findings of this study are available within the paper.
